# Changes in mental health of Korean adolescents before and during the COVID-19 pandemic: a special report using the Korea Youth Risk Behavior Survey

**DOI:** 10.4178/epih.e2023019

**Published:** 2023-02-14

**Authors:** Bomi Park, Jihee Kim, Jieun Yang, Sunhye Choi, Kyungwon Oh

**Affiliations:** 1 Department of Preventive Medicine, Chung-Ang University College of Medicine, Seoul, Korea; 2 Division of Health and Nutrition Survey and Analysis, Bureau of Chronic Disease Prevention and Control, Korea Disease Control and Prevention Agency, Cheongju, Korea

**Keywords:** Health surveys, Mental health, Adolescent, COVID-19

## Abstract

**OBJECTIVES:**

We aimed to study the effects of the coronavirus disease 2019 (COVID-19) pandemic on adolescents’ mental health in Korea.

**METHODS:**

We used data from the Korea Youth Risk Behavior Survey 2018-2021 with 227,139 students aged 12-18 years. We estimated the differences in depressive symptoms, suicidal ideation, and stress perception before (2018-2019) and during (2020-2021) the pandemic, as well as before (2019), the first year (2020) of, and the second year (2021) of the pandemic. We also examined whether COVID-19 is statistically associated with mental health.

**RESULTS:**

In both male and female adolescents, the prevalence of depressive symptoms, suicidal ideation, and stress perception was higher in the “not living with family,” “low household economic status,” and “self-rated unhealthy status” subgroups. The prevalence of depressive symptoms and stress perception was higher in middle school students. Adolescents were less likely to experience depressive symptoms (adjusted odds ratio [aOR], 0.86; 95% confidence interval [CI], 0.83 to 0.89), suicidal ideation (aOR, 0.80; 95% CI, 0.76 to 0.83), and stress perception (aOR, 0.76; 95% CI, 0.74 to 0.79) in 2020 than in 2019. However, there were more depressive symptoms (aOR, 1.06; 95% CI, 1.02 to 1.09), suicidal ideation (aOR, 1.15; 95% CI, 1.10 to 1.21), and stress perception (aOR, 1.19; 95% CI, 1.16 to 1.23) in 2021 than in 2020.

**CONCLUSIONS:**

The COVID-19 pandemic had positive impacts on mental health of adolescents in its early stages but has had negative impacts as the pandemic continues. Attention should be paid to adolescents who are particularly vulnerable to the mental health effects of the pandemic.

## INTRODUCTION

Since the coronavirus disease 2019 (COVID-19) was first reported in Wuhan, China in December 2019, COVID-19 has spread worldwide; and on March 11, 2020, the World Health Organization (WHO) declared COVID-19 to be a global pandemic [1]. Korea implemented infection control policies such as social distancing, travel restrictions, and quarantine to respond to the pandemic. This included restrictions on school attendance and conducting online classes over a long period of time which have significantly changed adolescents’ daily lives [2,3]. Adolescents spent less time with friends and on leisure activities outside their homes; they also had increased time spent at home with parents who worked from home, and were increasingly worried about their own and their family’s health and future [4]. Previous studies have reported that such changes affected adolescents’ learning activities and increased the use of digital media [5,6].

It has been reported that changes in daily life caused by COVID-19 acted as a complex and multifaceted psychosocial stressor, and that there were increased negative symptoms related to mental health such as depression, anxiety, and increased stress in adults during the COVID-19 pandemic [7,8]. In particular, adolescence is a time period during which rapid physical and mental development occurs, and adolescents are emotionally unstable and more sensitive to stress, emotional conflict, fear, and sudden changes in daily life compared to adults. Therefore, adolescents may be more vulnerable to the mental health effects of the COVID-19 pandemic [9]. In fact, previous studies have reported that the prevalence of anxiety, depression, and stress increased in adolescents during the COVID-19 pandemic [10-12]. Since the mental health status of adolescence is known to affect their ability to overcome difficulties later in adulthood [13], it is very significant to determine whether or not the mental health status of adolescents has changed during the COVID-19 pandemic from a life course perspective.

In addition, the COVID-19 pandemic has been shown to have more negative impacts on the mental health of vulnerable groups such as socioeconomically underprivileged population and female students [14,15]. Because there is a possibility that among adolescents there may be vulnerable groups whose mental health is more affected by the COVID-19 pandemic, it is important to identify vulnerable groups to provide effective interventions for the mental health of adolescents during the pandemic.

Most of the previous studies on the impact of COVID-19 on the mental health of adolescents have analyzed changes in the first year of the COVID-19 pandemic, but studies analyzing the impact of COVID-19 as it lasted for more than one year are scarce [16]. Therefore, this study aimed to investigate the effects of the COVID-19 pandemic on the mental health of adolescents over two years. In addition, we hypothesized that as the COVID-19 pandemic continues, the short-term effects of COVID-19 on the mental health of adolescents in the first year of the COVID-19 pandemic might be different from those in its second year. This study aimed to investigate changes in the mental health of adolescents by comparing the effects of COVID-19 on the mental health of adolescents before (2018-2019), the first year (2020), and the second year (2021) of COVID-19 as COVID-19 pandemic continued. Using subgroup analyses, this study also attempted to investigate the difference in the effects of the COVID-19 pandemic on the mental health of adolescents according to the characteristics of the participants, and to identify vulnerable groups.

## MATERIALS AND METHODS

### Study population

This study was conducted on 227,139 students in middle schools and high schools who participated in the 2018-2021 Korea Youth Risk Behavior Survey (KYRBS). The KYRBS is an anonymous, self-reported online survey of first–third-grade students in middle and high schools nationwide conducted annually by the Korea Disease Control and Prevention Agency (KDCA) in collaboration with the Ministry of Education that evaluates health behaviors among Korean adolescents. To produce health indicators representative of the Korean adolescents, a two-stage cluster sampling method was used to extract nationally representative students from 800 sampled schools (400 middle schools, and 400 high schools). Schools were selected as primary sampling units (PSUs), and classrooms within PSUs were selected as secondary sampling units (SSUs) [17].

### Measurements

To compare mental health in adolescents before and during the COVID-19 pandemic, the 2018 and 2019 years were classified as ‘before the COVID-19 pandemic’, and the 2020 and 2021 years were classified as ‘during the COVID-19 pandemic’. Additionally, to evaluate the effects of the prolonged COVID-19 pandemic, the 2020 year was defined as ‘the first year of the COVID-19 pandemic’, and the 2021 year was defined as ‘the second year of the COVID-19 pandemic’.

The mental health indicators in adolescents included the experience of depressive symptoms, suicidal ideation, and stress perception. The experience of depressive symptoms was defined as responding yes to the question: “During the past 12 months, have you ever felt so sad or hopeless almost every day for 2 weeks in a row that you stopped doing some usual daily activities?” Suicidal ideation was defined as responding yes to the question: “Have you seriously considered attempting suicide during the past 12 months?” Stress perception was defined as responding with “I feel a great deal of stress” or “I feel a lot of stress” among the response examples: I feel a great deal of stress, I feel a lot of stress, I feel a little stress, I do not feel much stress, I feel no stress” to the question: “How much stress do you usually feel?”

The demographic characteristics of the participants included sex (male and female students) and school level (high and middle school). The socioeconomic and health-related factors included residence type, household economic status, worsened household economic status after COVID-19, and self-rated health status. When asked about current residence type, those who responded that they were living with their family were classified as those living with their family, and those who responded that they were living in a relative’s house or a boarding house, living alone (including living with friends), living in a dormitory or a childcare facility (orphanage, social welfare facility, orphanage) were classified as not living with their family. In terms of household economic status, household economic status perceived by students was surveyed using five response examples: upper, upper-middle, middle, lower-middle, and lower household economic status, and was reclassified as high (upper, upper-middle), middle (middle), and low (lower-middle, lower). Worsened household economic status after COVID-19 was divided into responding yes (strongly agree, agree) and responding no (disagree, strongly disagree) based on the participant’s response to the question asking whether their household economic status was worse than before because of COVID-19. Self-rated health status was defined as being healthy (very healthy or healthy), normal (normal), or unhealthy (unhealthy or very unhealthy) based on the participant’s response to the question asking how they feel about their health status in general. Changes in household economic status after COVID-19 were surveyed only in 2020 and 2021, and the other variables were measured in 2018-2021.

### Statistical analysis

To calculate estimates representative of Korean adolescents, all statistical analyzes were performed considering the complex sample design. The general characteristics of the participants in the KYRBS in 2018-2021 were compared using PROC SURVEYFREQ. Mental health indicators were estimated as percentage and standard error (SE) using PROC SURVEYMEANS. After adjusting for school level, testing for the significance of a difference in mental health indicators between the before and during the COVID-19 pandemic time periods was performed using PROC SURVEYLOGISTIC. To identify vulnerable groups, the participants were analyzed by sex, school level, residence type, household economic status, worsened household economic status after COVID-19, and self-rated health status. Changes in mental health in adolescents before and during the COVID-19 pandemic, and in the first and second years of the COVID-19 pandemic were presented as adjusted odds ratios (aOR) using PROC SURVEYLOGISTIC. Covariates included sex, school level, residence type, household economic status, worsened household economic status after COVID-19, and self-rated health status. Analyses were performed for all participants and subgroups. All statistical analyses were performed using SAS version 9.4 (SAS Institute Inc., Cary, NC, USA), and the results were considered statistically significant if a p-value was < 0.05 after a two-tailed test.

### Ethics statement

The KYRBS was conducted without an institutional review board review in accordance with the Enforcement Decree of the Bioethics and Safety Act.

## RESULTS

The participants of this study were a total of 227,139 students in middle school and high school who participated in the 2018-2021 KYRBS. The numbers of the subjects who participated in the KYRBS before (2018-2019) and during (2020-2021) the COVID-19 pandemic were 117,343 (male: 52.0%, middle school students: 47.1%), and 109,796 (male: 51.8%, middle school students: 50.3%), respectively. The demographic and socioeconomic characteristics and self-rated health status of the adolescents who participated in the corresponding year’s KYRBS are shown in Table 1.

The prevalence of suicidal ideation and stress perception in male students were lower during the COVID-19 pandemic (2020-2021) compared to before the COVID-19 pandemic (2018-2019), and the subgroup analyses according to school level, residence type, household economic status, and self-rated health status also showed similar trends. There was no statistically significant difference in depressive symptoms in male students before and during the COVID-19 pandemic. The prevalence of depressive symptoms, suicidal ideation, and perceived stress in female students were all lower during the COVID-19 pandemic compared to before the COVID-19 pandemic, and the subgroup analysis also showed similar results. The prevalence of depressive symptoms, suicidal ideation, and stress perception in both male and female students were higher in those not living with their family, who had low household economic status, and who perceived themselves to be unhealthy, and the prevalence of depressive symptoms and stress perception were higher in high school students compared to middle school students. These trends were similar both before and during the COVID-19 pandemic (Table 2).

When we analyzed the prevalence of mental health problems in before (2019), the first year (2020), and the second year (2021), the prevalence of depressive symptoms (male: 22.2%, female: 34.6% in 2019; male: 20.1%, female: 30.7% in 2020), suicidal ideation (male: 9.4%, female: 17.1% in 2019; male: 8.1%, female: 13.9% in 2020), and stress perception (male: 31.7%, female: 48.8% in 2019; male: 28.1%, female: 40.7% in 2020) in male and female students decreased in the first year of the COVID-19 pandemic compared to before the pandemic. Meanwhile, the prevalence of depressive symptoms (male: 20.1%, female: 30.7% in 2020, 22.4% male, 31.4% female in 2021), suicidal ideation (male: 8.1%, female: 13.9% in 2020; male: 9.5%, female: 16.1% in 2021), and stress perception (male: 28.1%, female: 40.7% in 2020; male: 32.3%, female: 45.6% in 2021) in male and female students increased in the second year of the COVID-19 pandemic compared to the first year. In addition, when divided into subgroups according to school level, residence type, household economic status, worsened household economic status after COVID-19, and self-rated health status, the results showed that the prevalence of depressive symptoms, suicidal ideation, and stress perception in most subgroups was higher in 2021 compared to 2020 (Table 3).

The results of analyzing changes in mental health in adolescents during the first year of the COVID-19 pandemic found that: the prevalence of depressive symptoms (aOR, 0.86; 95% confidence interval [CI], 0.83 to 0.89), suicidal ideation (aOR, 0.80; 95% CI, 0.76 to 0.83), and stress perception (aOR, 0.76; 95% CI, 0.74 to 0.79) was significantly decreased in the first year of the COVID-19 pandemic compared to before the COVID-19 pandemic, and the results of analyzing subgroups according to sex or household economic status also showed similar results. In addition, the prevalence of stress perception was found to be significantly decreased in female students compared to male students, and there were no significant differences between the subgroups in the other cases (Table 4).

The results of analyzing the effects of the prolonged COVID-19 pandemic on mental health revealed that the prevalence of depressive symptoms (aOR, 1.06; 95% CI, 1.02 to 1.09), suicidal ideation (aOR, 1.15; 95% CI, 1.10 to 1.21), and stress perception (aOR, 1.19; 95% CI, 1.16 to 1.23) was higher in the second year of the COVID-19 pandemic compared to its first year. The results of analyzing subgroups according to sex, household economic status, and a worsened household economic status after COVID-19 also showed mostly similar trends. The prevalence of depressive symptoms in male students significantly increased compared to female students, and the prevalence of stress perception significantly increased in those having high household economic status compared to those not having high household economic status, and there were no significant differences between the groups in the other cases (Table 5).

## DISCUSSION

**Figure 1.**
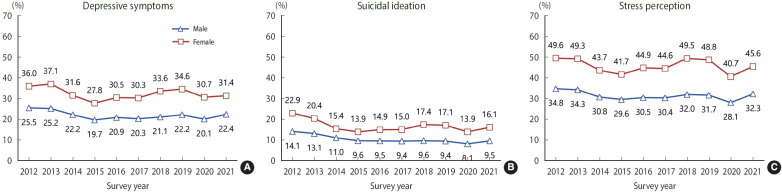
The prevalence of depressive symptoms, suicidal ideation, and stress perception by sex from 2012 to 2021 in the Korea Youth Risk Behavior Survey.

The results of analyzing data from KYRBS, a nationally representative health survey of Korean adolescents that is conducted annually, revealed that the prevalence of depressive symptoms, suicidal ideation, and stress perception in adolescents had shown a decreasing or stable trend since 2012 (Figure 1, Supplementary Material 1). However, the prevalence of depressive symptoms, suicidal ideation, and stress perception significantly decreased in 2020 (the first year of the COVID-19 pandemic) compared to before the COVID-19 pandemic; the health indicators deteriorated in the second year (2021) of the COVID-19 pandemic compared to its first year (2020), and the indicators tended to return to their pre-COVID-19 levels. In addition, the prevalence of depressive symptoms, suicidal ideation, and stress perception was higher in female students, those having low household economic status, those having worsened household economic status after COVID-19, and those who perceiving themselves to be unhealthy. However, the subgroups that were more vulnerable to mental health problems before COVID-19 were not more affected by the COVID-19 pandemic.

To prevent the spread of the COVID-19 outbreak, schools were closed and student activities were restricted, causing adolescents to become socially isolated. Some previous studies have reported that such changes in daily life during the COVID-19 pandemic worsened mental health, causing increased depression, anxiety, and suicidality [16,18,19]. Meanwhile, a previous study involving American adolescents reported that changes in daily activity, such as not going to school, not participating in after-school activities, and not exercising because of the COVID-19 pandemic did not affect depressive symptoms or anxiety [14]. This study found that the prevalence of depressive symptoms, suicidal ideation, and stress perception was lower in the first year of the COVID-19 pandemic compared to pre-COVID-19 levels. The contradictory results between the studies may be due to differences in social distancing policies, adolescents’ life patterns, and cultures between countries. As the first COVID-19 case in Korea was reported in January 2020, the opening of the first semester of the 2020 school year was delayed in accordance with the government’s policy to prevent the spread of COVID-19. Beginning in April 2020, online classes and school attendance were conducted sequentially by grade. Since the 2020 KYRBS was conducted between August and November 2020, the survey results might reflect the mental health status of adolescents at the time when the new semester for schools was postponed, and both online and offline classes were conducted in parallel. In other words, the results of this study suggest that, due to school closure and online classes in the first year of the COVID-19 pandemic, adolescents might have temporarily experienced reduced stress from peer relationships and studies, have increased sleep sufficiency, and have increased time spent with family, thereby reducing their levels of depressive symptoms, suicidal ideation, and stress perception [3].

Meanwhile, the results of this study found that the prevalence of depressive symptoms, suicidal ideation, and stress perception were higher in 2021 compared to 2020. Similar to the results of this study, the results of an online survey involving children and adolescents aged from 9 years to 24 years showed that the survey participants experienced more anxiety, anger, fear, and stress due to COVID-19 in 2021 compared to 2020, and that their experience of positive emotions such as interest and gratitude were decreased. In addition, such phenomenon was more prominent in high-risk adolescents [20]. Since the 2021 KYRBS was conducted from August to November 2021, the results of the 2021 KYRBS were likely to reflect the mental health status of adolescents in a prolonged COVID-19 situation. In Korea, beginning in the second semester of 2020, the stages of social distancing have been adjusted according to the number of confirmed COVID-19 cases, and this has caused continuous changes in the principles of school attendance. Although there were differences in physical attendance at schools depending on region and school conditions, students, except for third-grade students in high schools, went to school every other week or every other day in the first semester of 2021, and social isolation, loneliness, and irregular life patterns thus continued. The results of this study suggest that the prolonged COVID-19 pandemic and the resulting uncertainty and social isolation might deepen adolescents’ negative emotions, such as fear of infection, stress, and worries about their studies and career. If the COVID-19 pandemic continues, it is likely to have greater negative effects on mental health in adolescents. Therefore, as the COVID-19 pandemic is prolonged, further studies on the effects of the pandemic on mental health and related factors in adolescents are needed.

Several studies have reported that the COVID-19 pandemic did not have the same impact on all population groups, and that there were vulnerable groups of people who are more affected by the pandemic [4], including female students, and groups of people with low education levels, low household economic status, a small living space, and underlying mental illness [5,8,21]. This study also found that those who were not living with their family, had low household economic status, had worsened household economic status after COVID-19, and perceived themselves as unhealthy were more vulnerable to the effects of COVID-19. These findings suggest that intensive interventions should be provided to vulnerable groups to reduce the mental health burden caused by the COVID-19 pandemic.

This study differs from many previous studies in that it investigated the effects of the prolonged COVID-19 pandemic on the mental health of adolescents by analyzing data over two years after the COVID-19 outbreak. In addition, because the KYRBS was conducted on extracted samples to calculate estimates representative of Korean adolescents, and is conducted using the same methodology every year, this study could compare the prevalence trends of mental health by year. However, although this study analyzed yearly trends using data over several years, it was analyzed based on cross-sectional survey data. Therefore, there is a limitation in that it was not able to analyze changes in mental health over time at an individual level. In addition, although it is known that those with poor pre-existing mental health conditions are more affected by the COVID-19 pandemic [16], this study could not consider it in the analysis because information on pre-existing mental health conditions was not available.

## CONCLUSION

In the early stage of the COVID-19 pandemic, the mental health of adolescents tended to improve. However, as the COVID-19 pandemic continues, the prevalence of depressive symptoms, suicidal ideation, and stress perception in adolescents were significantly increased. In addition, the prevalence of depressive symptoms, suicidal ideation, and stress perception were generally higher in adolescents who were not living with their family, had low household economic status, and perceived their health as unhealthy. Understanding the long-term impact of the COVID-19 pandemic on the mental health of adolescents is important for formulating public health strategies to promote the mental health of adolescents during the COVID-19 pandemic.

## Figures and Tables

**Table 1.** Characteristics of participants before (2018-2019) and during COVID-19 (2020-2021) in the Korea Youth Risk Behavior Survey

Characteristics		Before COVID-19		During COVID-19		Before COVID-19	During COVID-19	p-value
		2018	2019	2020	2021	2018-2019	2020-2021	
Total^1^		60,040	57,303	54,948	54,848	117,343	109,796	
Sex								0.843
	Male	30,463 (52.1)	29,841 (52.0)	28,353 (51.9)	28,401 (51.7)	60,304 (52.0)	56,754 (51.8)	
	Female	29,577 (47.9)	27,462 (48.0)	26,595 (48.1)	26,447 (48.3)	57,039 (48.0)	53,042 (48.2)	
School level								<0.001
	Middle school	30,229 (46.4)	29,384 (47.9)	28,961 (49.6)	30,015 (51.0)	59,613 (47.1)	58,976 (50.3)	
	High school	29,811 (53.6)	27,919 (52.1)	25,987 (50.4)	24,833 (49.0)	57,730 (52.9)	50,820 (49.7)	
Residence type								0.003
	With family	56,654 (95.2)	54,267 (95.4)	52,332 (96.2)	52,426 (96.2)	110,921 (95.3)	104,758 (96.2)	
	No family	3,386 (4.8)	3,036 (4.6)	2,616 (3.8)	2,422 (3.8)	6,422 (4.7)	5,038 (3.8)	
Household economic status								<0.001
	High	24,207 (40.8)	22,505 (39.7)	21,339 (39.9)	21,568 (40.1)	46,712 (40.3)	42,907 (40.0)	
	Middle	27,808 (46.0)	27,457 (47.8)	26,397 (47.5)	27,077 (49.0)	55,265 (46.9)	53,474 (48.2)	
	Low	8,025 (13.2)	7,341 (12.5)	7,212 (12.6)	6,203 (10.9)	15,366 (12.9)	13,415 (11.8)	
Worsened household economic status after COVID-19								NA
	No	NA	NA	38,109 (70.0)	38,136 (70.1)	NA	76,245 (70.1)	
	Yes	NA	NA	16,839 (30.0)	16,712 (29.9)	NA	33,551 (29.9)	
Self-rated health status								<0.001
	Healthy	43,300 (71.6)	40,256 (70.0)	38,444 (69.6)	35,529 (64.7)	83,556 (70.8)	73,973 (67.2)	
	Normal	12,825 (21.6)	12,810 (22.6)	12,342 (22.6)	14,298 (26.1)	25,635 (22.1)	26,640 (24.4)	
	Unhealthy	3,915 (6.7)	4,237 (7.5)	4,162 (7.7)	5,021 (9.2)	8,152 (7.1)	9,183 (8.5)	

Values are presented as number (weighted %). COVID-19, coronavirus disease 2019; NA, not available.

1 Categories may not add up to total number due to missing data.

**Table 2.** Prevalence of mental health by participants’ characteristics before and during COVID-19 in the Korea Youth Risk Behavior Survey^1^

Characteristics			Depressive symptoms			Suicidal ideation			Stress perception		
			2018-2019	2020-2021	p-value^2^	2018-2019	2020-2021	p-value^2^	2018-2019	2020-2021	p-value^2^
Male											
	Total		21.6 (0.2)	21.2 (0.2)	0.486	9.5 (0.1)	8.8 (0.1)	0.001	31.9 (0.2)	30.2 (0.2)	<0.001
	School level										
		Middle school	19.6 (0.3)	19.8 (0.3)	0.300	9.4 (0.2)	8.7 (0.2)	0.022	29.4 (0.3)	28.3 (0.3)	0.013
		High school	23.5 (0.3)	22.7 (0.3)	0.074	9.5 (0.2)	8.9 (0.2)	0.021	34.1 (0.3)	32.1 (0.3)	<0.001
	Residence type										
		With family	21.3 (0.2)	21.0 (0.2)	0.869	9.2 (0.1)	8.6 (0.1)	0.006	31.5 (0.2)	30.0 (0.2)	<0.001
		No family	28.6 (0.9)	25.8 (1.0)	0.066	15.6 (0.7)	13.6 (0.8)	0.100	39.0 (0.9)	34.1 (1.0)	0.000
	Household economic status										
		High	20.5 (0.3)	20.4 (0.3)	0.678	8.5 (0.2)	8.0 (0.2)	0.149	29.0 (0.3)	28.5 (0.3)	0.472
		Middle	20.2 (0.3)	19.8 (0.3)	0.427	8.5 (0.2)	8.0 (0.2)	0.080	31.2 (0.3)	28.7 (0.3)	<0.001
		Low	30.7 (0.6)	29.7 (0.6)	0.297	16.6 (0.4)	14.7 (0.4)	0.001	44.2 (0.6)	41.8 (0.6)	0.003
	Self-rated health status										
		Healthy	18.7 (0.2)	18.4 (0.2)	0.628	7.2 (0.1)	6.8 (0.1)	0.031	26.5 (0.2)	24.7 (0.2)	<0.001
		Normal	28.3 (0.5)	26.0 (0.4)	0.003	14.0 (0.3)	11.7 (0.3)	<0.001	44.9 (0.5)	39.7 (0.5)	<0.001
		Unhealthy	40.9 (0.9)	35.5 (0.8)	0.000	26.1 (0.8)	20.8 (0.7)	<0.001	64.0 (0.8)	57.4 (0.8)	<0.001
Female											
	Total		34.1 (0.2)	31.1 (0.2)	<0.001	17.3 (0.2)	15.0 (0.2)	<0.001	49.2 (0.3)	43.2 (0.3)	<0.001
	School level										
		Middle school	33.0 (0.3)	29.4 (0.3)	<0.001	18.9 (0.3)	15.1 (0.3)	<0.001	45.4 (0.4)	38.9 (0.3)	<0.001
		High school	35.1 (0.3)	32.8 (0.4)	<0.001	15.9 (0.3)	14.9 (0.3)	0.009	52.5 (0.4)	47.5 (0.4)	<0.001
	Residence type										
		With family	33.8 (0.2)	30.9 (0.2)	<0.001	17.1 (0.2)	14.7 (0.2)	<0.001	48.9 (0.3)	42.9 (0.3)	<0.001
		No family	39.2 (1.1)	36.7 (1.2)	0.085	21.1 (0.9)	22.4 (1.0)	0.615	54.3 (1.2)	49.9 (1.2)	0.018
	Household economic status										
		High	32.1 (0.3)	29.1 (0.3)	<0.001	15.4 (0.3)	13.3 (0.3)	<0.001	46.0 (0.4)	40.0 (0.4)	<0.001
		Middle	32.6 (0.3)	30.0 (0.3)	<0.001	15.9 (0.2)	13.9 (0.2)	<0.001	47.9 (0.3)	42.4 (0.3)	<0.001
		Low	45.4 (0.6)	42.5 (0.6)	0.001	27.7 (0.5)	25.5 (0.6)	0.001	62.8 (0.6)	56.7 (0.6)	<0.001
	Self-rated health status										
		Healthy	27.6 (0.3)	24.9 (0.3)	<0.001	12.1 (0.2)	10.3 (0.2)	<0.001	40.4 (0.3)	34.2 (0.3)	<0.001
		Normal	41.8 (0.5)	37.0 (0.4)	<0.001	22.5 (0.4)	18.6 (0.3)	<0.001	60.6 (0.4)	53.0 (0.4)	<0.001
		Unhealthy	58.8 (0.7)	52.6 (0.7)	<0.001	39.4 (0.8)	34.3 (0.7)	<0.001	79.5 (0.6)	71.4 (0.6)	<0.001

Values are presented as weighted % (standard error). COVID-19, coronavirus disease 2019.

1 All results are adjusted for grade.

2 p-value is for the difference of prevalence of mental health between before COVID-19 (2018-2019) and during COVID-19 (2020-2021).

**Table 3.** Prevalence of mental health by participants’ characteristics in before, the first year of, and the second year of COVID-19 in the Korea Youth Risk Behavior Survey^1^

Characteristics			Depressive symptoms			Suicidal ideation			Stress perception		
			2019	2020	2021	2019	2020	2021	2019	2020	2021
Male											
	Total		22.2 (0.3)	20.1 (0.3)	22.4 (0.3)	9.4 (0.2)	8.1 (0.2)	9.5 (0.2)	31.7 (0.3)	28.1 (0.3)	32.3 (0.3)
	School level										
		Middle school	20.1 (0.4)	17.8 (0.4)	21.7 (0.4)	9.4 (0.3)	7.4 (0.2)	10.0 (0.3)	29.1 (0.4)	24.9 (0.4)	31.5 (0.5)
		High school	24.1 (0.4)	22.2 (0.4)	23.1 (0.4)	9.3 (0.3)	8.8 (0.3)	9.0 (0.3)	34.1 (0.4)	31.1 (0.5)	33.2 (0.4)
	Residence type										
		With family	21.8 (0.3)	19.9 (0.3)	22.2 (0.3)	9.0 (0.2)	7.9 (0.2)	9.3 (0.2)	31.3 (0.3)	27.9 (0.3)	32.2 (0.3)
		No family	29.9 (1.2)	24.1 (1.2)	27.5 (1.6)	16.2 (1.0)	12.7 (1.0)	14.5 (1.4)	40.9 (1.2)	32.0 (1.4)	36.1 (1.4)
	Household economic status										
		High	21.1 (0.4)	18.7 (0.4)	22.1 (0.4)	8.1 (0.3)	7.1 (0.2)	8.9 (0.3)	28.7 (0.4)	25.6 (0.5)	31.3 (0.5)
		Middle	20.7 (0.4)	18.9 (0.4)	20.7 (0.4)	8.5 (0.3)	7.5 (0.3)	8.5 (0.3)	31.2 (0.4)	27.1 (0.4)	30.3 (0.4)
		Low	31.7 (0.8)	28.7 (0.8)	30.9 (0.9)	17.1 (0.7)	13.5 (0.6)	15.9 (0.7)	44.1 (0.8)	39.6 (0.8)	44.3 (0.9)
	Worsened household economic status after COVID-19										
		No	NA	18.1 (0.3)	20.7 (0.3)	NA	7.1 (0.2)	8.4 (0.2)	NA	25.8 (0.4)	29.7 (0.4)
		Yes	NA	24.7 (0.5)	26.3 (0.5)	NA	10.3 (0.3)	12.0 (0.4)	NA	33.4 (0.6)	38.2 (0.5)
	Self-rated health status										
		Healthy	19.3 (0.3)	17.5 (0.3)	19.4 (0.3)	7.0 (0.2)	6.4 (0.2)	7.2 (0.2)	26.4 (0.3)	23.3 (0.3)	26.3 (0.3)
		Normal	28.3 (0.6)	24.6 (0.7)	27.2 (0.6)	14.1 (0.5)	10.8 (0.5)	12.5 (0.4)	44.3 (0.7)	38.0 (0.7)	41.3 (0.6)
		Unhealthy	40.8 (1.2)	36.0 (1.1)	35.1 (1.1)	26.3 (1.1)	19.8 (1.0)	21.6 (0.9)	62.3 (1.2)	54.5 (1.2)	59.6 (1.1)
Female											
	Total		34.6 (0.4)	30.7 (0.3)	31.4 (0.3)	17.1 (0.3)	13.9 (0.3)	16.1 (0.3)	48.8 (0.4)	40.7 (0.4)	45.6 (0.4)
	School level										
		Middle school	34.1 (0.5)	28.4 (0.4)	30.4 (0.4)	19.2 (0.4)	13.3 (0.3)	16.9 (0.4)	45.9 (0.5)	36.2 (0.4)	41.5 (0.5)
		High school	35.1 (0.5)	33.0 (0.5)	32.5 (0.5)	15.3 (0.4)	14.5 (0.4)	15.3 (0.4)	51.5 (0.5)	45.2 (0.6)	49.9 (0.5)
	Residence type										
		With family	34.5 (0.4)	30.5 (0.4)	31.2 (0.3)	17.0 (0.3)	13.7 (0.3)	15.8 (0.3)	48.6 (0.4)	40.5 (0.4)	45.4 (0.4)
		No family	37.8 (1.6)	35.5 (1.4)	38.0 (1.9)	19.9 (1.3)	19.4 (1.3)	25.7 (1.6)	52.5 (1.8)	46.8 (1.6)	53.4 (1.7)
	Household economic status										
		High	32.9 (0.5)	28.7 (0.5)	29.4 (0.5)	15.4 (0.4)	12.4 (0.4)	14.1 (0.4)	46.1 (0.6)	37.0 (0.6)	43.0 (0.5)
		Middle	32.9 (0.5)	29.6 (0.4)	30.4 (0.4)	15.7 (0.3)	12.6 (0.3)	15.1 (0.4)	47.5 (0.5)	39.8 (0.5)	45.0 (0.5)
		Low	46.0 (0.8)	41.3 (0.8)	43.8 (1.0)	27.9 (0.8)	23.4 (0.8)	28.0 (0.9)	61.5 (0.8)	55.3 (0.8)	58.4 (0.9)
	Worsened household economic status after COVID-19										
		No	NA	27.6 (0.4)	28.3 (0.4)	NA	12.0 (0.3)	13.9 (0.3)	NA	37.8 (0.4)	42.4 (0.4)
		Yes	NA	37.9 (0.4)	39.0 (0.6)	NA	18.2 (0.5)	21.5 (0.5)	NA	47.4 (0.6)	53.6 (0.6)
	Self-rated health status										
		Healthy	28.4 (0.4)	25.2 (0.4)	24.7 (0.4)	12.2 (0.3)	9.7 (0.2)	11.0 (0.3)	40.0 (0.4)	32.3 (0.4)	36.2 (0.4)
		Normal	41.6 (0.7)	36.9 (0.6)	37.1 (0.6)	21.6 (0.5)	17.6 (0.5)	19.5 (0.5)	59.1 (0.6)	51.0 (0.6)	54.8 (0.6)
		Unhealthy	56.8 (1.0)	52.2 (1.0)	53.0 (0.9)	38.0 (1.1)	32.7 (1.0)	35.7 (0.9)	78.4 (0.8)	70.0 (0.9)	72.7 (0.9)

Values are presented as weighted % (standard error). COVID-19, coronavirus disease 2019; NA, not available.

1 All results are adjusted for grade.

**Table 4.** Odd ratios^1^ of mental health in the first year of COVID-19 (2020) compared to before COVID-19 (2019) by participants’ characteristics in the Korea Youth Risk Behavior Survey

Variables	Depressive symptoms	Suicidal ideation	Stress perception
Total^2^	0.86 (0.83, 0.89)	0.80 (0.76, 0.83)	0.76 (0.74, 0.79)
Sex^3^			
Male	0.87 (0.83, 0.92)	0.84 (0.78, 0.89)	0.82 (0.79, 0.86)
Female	0.84 (0.81, 0.88)	0.77 (0.73, 0.82)	0.72 (0.69, 0.75)
Household economic status^4^			
High	0.84 (0.80, 0.88)	0.81 (0.76, 0.87)	0.76 (0.72, 0.79)
Middle	0.87 (0.83, 0.91)	0.80 (0.75, 0.85)	0.76 (0.73, 0.79)
Low	0.85 (0.79, 0.91)	0.77 (0.71, 0.84)	0.80 (0.75, 0.85)

Values are presented as adjusted odds ratio (95% confidence interval). COVID-19, coronavirus disease 2019.

1 Adjusted odds ratio was calculated from multiple logistic regression analysis with complex sampling.

2 Adjusted for sex, school level, household economic status, residential type, and self-rated health status.

3 Adjusted for school level, household economic status, residential type, and self-rated health status.

4 Adjusted for sex, school level, residential type, and self-rated health status.

**Table 5.** Odd ratios^1^ of mental health conditions in the second year of COVID-19 (2021) compared to the first year of COVID-19 (2020) by participants’ characteristics in the Korea Youth Risk Behavior Survey

Variables	Depressive symptoms	Suicidal ideation	Stress perception
Total^2^	1.06 (1.02, 1.09)	1.15 (1.10, 1.21)	1.19 (1.16, 1.23)
Sex^3^			
Male	1.12 (1.07, 1.18)	1.15 (1.07, 1.23)	1.19 (1.14, 1.24)
Female	1.01 (0.97, 1.05)	1.16 (1.10, 1.23)	1.19 (1.15, 1.24)
Household economic status^4^			
High	1.09 (1.04, 1.14)	1.16 (1.08, 1.24)	1.26 (1.21, 1.32)
Middle	1.04 (0.99, 1.08)	1.14 (1.07, 1.22)	1.16 (1.11, 1.20)
Low	1.05 (0.97, 1.13)	1.17 (1.06, 1.28)	1.11 (1.03, 1.19)
Worsened household economic status after COVID-19^5^			
Yes	1.03 (0.98, 1.09)	1.17 (1.09, 1.25)	1.21 (1.15, 1.27)
No	1.07 (1.03, 1.11)	1.15 (1.09, 1.21)	1.18 (1.14, 1.22)

Values are presented as adjusted odds ratio (95% confidence interval). COVID-19, coronavirus disease 2019.

1 Adjusted odds ratio was calculated from multiple logistic regression analysis with complex sampling.

2 Adjusted for sex, school level, household economic status, worsened household economic status after COVID-19, residential type, and self-rated health status.

3 Adjusted for school level, household economic status, worsened household economic status after COVID-19, residential type, and self-rated health status.

4 Adjusted for sex, school level, worsened household economic status after COVID-19, residential type, and self-rated health status.

5 Adjusted for sex, school level, household economic status, residential type, and self-rated health status.

## SUPPLEMENTARY MATERIALS

Korean version is available at https://www.e-epih.org/.

Supplementary Material 1. The prevalence of depressive symptoms, suicidal ideation, and stress perception from 2012 to 2021 in the Korea Youth Risk Behavior Survey
