# Supplementary material for: Changes in mental health of Korean adolescents before and during the COVID-19 pandemic: a special report using the Korea Youth Risk Behavior Survey
Source: Epidemiol Health. 2023 Feb 14;45:e2023019. doi: 10.4178/epih.e2023019 (PMC10581890; doi:10.4178/epih.e2023019)
Supplement: Supplementary file 1 [file epih-45-e2023019-Korean-Supplementary.docx]

Change in Mental health of Korean adolescents before and during the COVID-19 pandemic: a special report of the Korea Youth Risk Behavior Survey (KYRBS)

Running title: Adolescents’ mental health before and during COVID-19

Bomi Park^1*†^, Jihee Kim ^2*^, Jieun Yang^2^, Sunhye Choi^2^, Kyungwon Oh^2†^

* Bomi Park and Jihee Kim was contributed equally as co-first authors.

† Bomi Park and Kyungwon Oh was contributed equally as co-corresponding authors.

1 Department of Preventive Medicine, College of Medicine, University of Chung-Ang, Seoul, Korea

2 Division of Health and Nutrition Survey an Analysis, Bureau of Chronic Disease Prevention and Control, Korea Disease Control and Prevention Agency, Cheongju-si, Korea

Correspondence: Bomi Park

Department of Preventive Medicine, College of Medicine, Chung-Ang University, 84 Heukseouk-Ro, DongJak-Gu, Seoul, 06974, Korea

E-mail: bpark@cau.ac.kr

Co-Correspondence: Kyungwon Oh

Division of Health and Nutrition Survey and Analysis, Bureau of Chronic Disease Prevention and Control, Korea Disease Control and Prevention Agency, 187 Osongsaengmyeong 2-ro, Heungduk-gu, Cheongju 28159, Korea

E-mail: [kwoh27@korea.kr](mailto:kwoh27@korea.kr)

**Introduction**

2019년 12월에 중국 우한에서 첫 번째 코로나19 환자가 발생한 이후 코로나19는 전세계적으로 퍼졌으며, 2020년 3월 11일 세계보건기구는 팬데믹을 선포했다[1]. 우리나라는 코로나19 유행에 대응하기 위해 사회적 거리두기, 여행 제한, 격리 등의 정책을 시행하였고, 장기간 등교 제한, 온라인 수업 시행 등으로 인해 청소년의 일상 생활에도 많은 변화가 생겼다[2,3]. 청소년들은 친구들과 보내는 시간 및 집 밖에서의 여가 활동이 감소한 반면 재택 근무를 하는 부모와 집에서 보내는 시간이 증가하였고, 본인과 가족의 건강 및 미래에 대한 두려움이 커졌다[4]. 이러한 변화가 청소년들의 학습 활동에 영향을 미치고 디지털 미디어의 사용을 증가시켰다는 선행 연구결과가 있다[5,6].

코로나19로 인한 일상생활의 변화가 복잡하고 다면적인 심리사회적 스트레스 요인으로 작용하여 코로나19 이후 성인에서 우울, 불안, 스트레스 증가 등 정신건강과 관련한 부정적 증상이 증가하였다고 보고되었다[7,8], 그런데 특히 청소년기는 신체적, 정신적으로 급격한 발달이 일어나는 시기로 성인에 비해 감정적으로 불안하고 스트레스, 정서적 갈등, 두려움, 갑작스러운 일상생활의 변화를 더 민감하게 받아들이는 시기이므로 코로나19가 정신건강에 미치는 영향에 더 취약한 시기일 수 있다[9]. 실제로 코로나19 이후 청소년에서 불안, 우울, 스트레스 등이 증가했다는 연구 결과들이 보고되고 있다[10-12]. 청소년기의 정신건강 상태는 이후 성인기에 어려움을 극복하는 능력에 영향을 미친다고 알려져 있기 때문에[13], 코로나19 이후 청소년의 정신건강 상태의 변화 여부를 파악하는 것은 생애주기적으로 매우 중요한 의미가 있다.

또한, 코로나19 유행은 사회경제적으로 소외된 계층, 여학생 등 취약한 집단의 정신건강에 더 부정적 영향을 미치는 것으로 나타났다[14,15]. 청소년 중에서도 코로나19로 인해 정신건강에 더 많은 영향을 받는 취약한 집단이 있을 가능성이 있어 유행 이후 청소년의 정신건강을 효과적으로 중재하기 위해서는 취약 집단을 파악하는 것이 중요하다.

코로나19가 청소년의 정신건강에 미치는 영향에 관한 기존 연구의 대부분이 코로나19 이후 첫 1년 동안의 변화를 분석하였으며, 그 이후까지의 영향을 분석한 연구는 거의 없다[16]. 따라서 본 연구에서는 코로나19가 2년에 걸쳐 청소년 정신 건강에 미치는 영향을 살펴보고자 하였다. 또한 코로나19 유행이 시작하면서 유행 첫해에 단기적으로 청소년 정신건강에 미치는 영향은 코로나19 유행이 지속되면서 두번째 해에 청소년 정신건강에 미치는 영향과 다를 수 있다는 가설을 설정하였으며, 본 연구에서는 코로나19 전(2018-2019년), 유행 첫 번째 해(2020년), 유행 두 번째 해(2021년)를 비교하여 코로나19가 지속됨에 따라 청소년 정신건강의 변화를 살펴보고자 하였다. 또한 하위그룹별 분석을 통해서 대상자의 특성에 따라 코로나19 유행이 정신건강에 미치는 영향의 차이를 파악하고 취약계층을 확인하고자 하였다.

**Materials and Methods**

**Study population**

본 연구는 2018-2021년 청소년건강행태조사(The Korea Youth Risk Behavior Survey (KYRBS))에 참여한 중학교 1학년~고등학교 3학년 227,139명의 학생을 대상으로 수행하였다. 청소년건강행태조사는 우리나라 청소년의 건강행태를 파악하기 위해 전국 중학교 1학년부터 고등학교 3학년 학생을 대상으로 질병관리청이 교육부와 공동으로 매년 시행하고 있는 익명성 자기기입식 온라인 조사이다. 전국 청소년을 대표하는 건강지표를 산출하기 위해 2단계 층화집락추출법을 사용하여 800개 표본학교(중학교 400개, 고등학교 400개)에 재학중인 학생을 추출하였으며 1차 추출단위는 학교, 2차 추출단위는 학급이었다[17].

**Measurements**

코로나19 전후 청소년 정신건강을 비교하기 위해서 2018년과 2019년을 통합하여 코로나19 이전으로, 2020년과 2021년을 통합하여 코로나19 이후로 나누었으며, 코로나19 유행 지속에 의한 영향 여부를 평가하기 위해 2020년은 코로나19 유행 첫 번째 해, 2021년은 코로나19 유행 두 번째 해로 정의하였다.

청소년의 정신건강 지표로 우울감 경험, 자살 생각, 스트레스 인지를 포함하였다. 우울감 경험은 “최근 12개월 동안 2주 내내 일상생활을 중단할 정도로 슬프거나 절망감을 느낀 적이 있었습니까?” 질문에 대해 “없다 혹은 있다”의 응답보기 중 “있다”고 응답한 경우로 정의하였다. 자살 생각은 “최근 12개월 동안 심각하게 자살을 생각한 적이 있었습니까?” 라는 질문에 “없다 혹은 있다”의 응답보기 중 “있다”고 응답한 경우로 정의하였다. 스트레스 인지는 “평상시 스트레스를 얼마나 느끼고 있습니까” 라는 질문에 “대단히 많이 느낀다, 많이 느낀다, 조금 느낀다, 별로 느끼지 않는다, 전혀 느끼지 않는다”의 응답보기 중 “대단히 많이, 많이 느낀다”고 응답한 경우로 정의하였다.

인구학적 특징으로는 성별(남학생, 여학생)과 학교급(고등학생, 중학생)을 고려하였다. 사회경제적 및 신체적 요인으로는 거주 형태, 가정의 경제적 상태, 코로나19 이후 가정경제 상태 악화 여부, 주관적 건강 인지 상태를 고려하였다. 현재 거주 형태를 묻는 질문에 가족과 함께 살고 있다고 응답한 경우는 가족과 함께 거주하는 군으로 분류하였고 친척집 거주, 하숙, 자취(친구들과 같이 사는 경우 포함), 기숙사, 보육시설(고아원, 사회복지시설, 보육원)에 거주한다고 응답한 경우는 가족과 함께 살고 있지 않은 군으로 분류하였다. 가정의 경제적 상태는 학생이 주관적으로 생각하는 가정의 경제 수준을 상, 중상, 중, 중하, 하의 다섯 가지 응답보기로 조사하였고 높음(상, 중상), 중간(중), 낮음(중하, 하)으로 재분류하였다. 코로나19로 인해 학생 가정의 경제적 상태가 이전보다 어려워졌다고 생각하냐고 묻는 질문에 대한 답변을 이용하여 코로나19 이후 가정경제상태 악화 여부를 그렇다(매우 그렇다, 그런 편이다)와 그렇지 않다(그렇지 않은 편이다, 전혀 그렇지 않다)로 나누었다. 평상시 자신의 건강상태가 어떻다고 생각하냐는 설문에 대한 답변을 이용하여 주관적 건강 인지 상태를 건강함(매우 건강한 편이다 혹은 건강한 편이다), 보통(보통이다), 건강하지 못함(건강하지 못한 편이다 혹은 매우 건강하지 못한 편이다)으로 정의하였다. 이 중 코로나 19이후 가정경제상태 변화 여부는 2020, 2021년에만 조사되었으며, 나머지 변수는 2018-2021 년에 모두 측정되었다.

**Statistical analysis**

모든 통계 분석은 우리나라 청소년을 대표하는 추정치를 산출하기 위해 복합표본설계를 고려하였다. 2018~2021년 청소년건강행태조사에 참여한 대상자의 일반적인 특성은 PROC SURVEYFREQ을 이용하여 비교하였다. 정신건강 지표는 PROC SURVEYMEANS를 통해 %와 SE로 추정하였으며, 코로나19 유행 전과 유행시기 간 정신건강 지표 차이의 유의성 검정은 학년을 보정한 후 PROC SURVEYLOGISTIC을 통해 확인하였다. 취약집단을 파악하기 위해 조사 대상을 성별, 학년, 가족 동거여부, 가정 경제상태, 코로나19 이후 가정경제 악화 여부, 주관적 건강 상태별로 구분하여 분석하였다. 코로나19 유행 전과 유행 시기 및 코로나19 유행 첫번째 해와 두번째 해에 청소년 정신건강의 변화 여부는 PROC SURVEYLOGISTIC을 이용하여 Adjusted Odds Ratios로 제시하였다. 보정변수는 성별, 학년, 가족 동거여부, 가정 경제상태, 코로나19 이후 가정 경제 악화 여부, 주관적 건강 상태 등을 포함하였으며, 분석은 전체 대상자와 하위그룹에서 각각 수행하였다. 모든 통계 분석은 SAS version 9.4 (SAS Institute Inc., Cary, NC, USA)를 사용하여 수행하였으며, 양측검정 후 p-value<0.05 이면 결과가 통계적으로 유의한 것으로 판단하였다.

**Ethics statement**

청소년건강행태조사는 생명윤리 및 안전에 관한 법률 및 시행규칙에 근거하여 기관생명윤리위원회(IRB) 심의 없이 조사를 수행하였다.

**Results**

연구대상자는 2018년~2021년 청소년건강행태조사에 참여한 중학교 1학년~고등학교 3학년에 재학중인 총 227,139 명이었다. 코로나19 이전(2018-2019년)에 참여한 대상자는 117,343명(남자 52.0%, 중학생 47.1%), 코로나19 이후 (2020-2021년)에 참여한 대상자는 109,796명(남자 51.8%, 중학생 50.3%)이었다. 각 년도의 조사에 참여한 청소년의 인구사회경제적 특성과 주관적 건강상태는 표1에 제시하였다(Table 1).

코로나19 전(2018-2019년)에 비해 코로나19 이후(2020-2021년)에 남학생의 자살생각, 스트레스 인지는 더 낮았으며, 학년, 거주형태, 경제상태, 주관적 건강인지에 따른 하위그룹 분석에서도 유사한 경향이었다. 코로나19 유행 전후 남학생의 우울감 경험은 통계적으로 유의한 수준의 차이가 없었다. 코로나19 이전에 비해 이후 여학생의 우울감 경험, 자살 생각, 스트레스 인지 모두 낮았으며 하위그룹 분석에서도 유사하였다. 남녀 모두에서 가족과 함께 살지 않는 경우, 가정 경제 상태가 낮은 경우, 자신의 건강상태가 건강하지 않다고 인지하는 경우에 우울감, 자살 생각, 스트레스 인지율이 더 높았으며 고등학생이 중학생보다 우울감과 스트레스 인지율이 더 높았다. 이러한 경향은 코로나19 유행 전후 시기 모두에서 동일하였다(Table 2).

코로나19 유행 첫 번째 해(2020년)와 두 번째 해(2021년)를 구분하여 분석 시, 2019년에 비해 코로나19 유행 첫 번째 해인 2020년에 남녀 학생의 우울감(남자 22.2%, 여자 34.6% in 2019; 남자 20.1%, 여자 30.7% in 2020), 자살 생각(남자 9.4%, 여자 17.1% in 2019; 남자 8.1%, 여자 13.9% in 2020), 스트레스 인지(남자 31.7%, 여자 48.8% in 2019; 남자 28.1%, 여자 40.7% in 2020) 경험이 감소하였다. 반면, 코로나19 유행 첫 번째 해(2020년)에 비해 두 번째 해(2021년)에 남, 여학생의 우울감(남자 20.1%, 여자 30.7% in 2020; 남자 22.4%, 여자 31.4% in 2021), 자살 생각(남자 8.1%, 여자 13.9% in 2020; 남자 9.5%, 여자 16.1% in 2021), 스트레스 인지(남자 28.1%, 여자 40.7% in 2020; 남자 32.3%, 여자 45.6% in 2021) 경험이 증가하였다. 또한 학년, 거주형태, 가정경제상태, 코로나19 이후 가정경제 악화 여부, 주관적 건강상태에 따라 하위그룹으로 구분하였을 때, 대부분 하위 그룹에서 2021년의 우울감, 자살 생각, 스트레스 인지의 경험이 2020년에 비해 더 높았다(Table 3).

코로나19 유행 첫 1년간의 청소년 정신건강 변화를 분석한 결과, 코로나 유행 전(2019년)에 비해 코로나19 유행 첫 번째 해(2020년)에는 우울감 경험 (aOR:0.86, 95%CI 0.83-0.89), 자살 생각(aOR:0.80, 95%CI 0.76-0.83), 스트레스 인지(aOR:0.76, 95%CI 0.74-0.79)가 유의하게 감소하였고, 이는 성별 혹은 가정의 경제 상태에 따른 하위 그룹 별 분석결과에서도 유사하였다. 여학생이 남학생보다 스트레스 인지 경험률이 유의하게 낮아지는 것으로 보였으며 그 외의 경우에는 그룹 간에 유의한 차이가 없었다(Table 4).

코로나19 유행의 장기화가 정신건강에 미치는 영향 여부를 분석한 결과, 코로나19 유행 첫 번째 해(2020년)에 비해서 두 번째 해(2021년)에는 우울감 경험(aOR:1.06, 95%CI 1.02-1.09), 자살 생각(aOR:1.15, 95%CI 1.10-1.21), 스트레스 인지(aOR:1.19, 95%CI 1.16-1.23) 경험이 높았으며, 성별, 가정의 경제 상태, 코로나 19 이후 가정경제 악화 여부에 따른 하위 그룹별 분석에서도 대부분 유사한 경향이었다. 남학생이 여학생보다 우울감 경험률이 유의하게 높아졌고, 가정경제상태가 높은 경우가 그렇지 않은 경우 보다 스트레스 인지율이 유의하게 높아지는 것으로 보였으며 그 외의 경우에는 그룹 간 유의한 차이가 없었다(Table 5).

**Discussion**

우리나라 청소년을 대상으로 매년 반복적으로 수행된 국가 단위의 건강조사 자료를 이용하여 분석한 결과, 2012년부터 청소년의 우울감 경험, 자살 생각, 스트레스 인지는 감소 혹은 유지하는 추이였으나(Figure 1, Supplementary table 1) 코로나 19 유행 이후 첫 번째 해인 2020년에는 코로나 19 전에 비해 우울감 경험, 자살 생각, 스트레스 인지가 큰 폭으로 감소하였고, 코로나 19 유행 두 번째 해(2021년)에는 첫 번째 해(2020년)에 비해 정신건강 지표가 악화되어 코로나19 이전의 수준으로 돌아오는 경향을 보였다. 또한 여학생, 가정 경제상태가 낮은 경우, 코로나 19 이후 가정 경제 상태가 악화된 경우, 건강하지 않다고 스스로 인지하는 학생의 우울감 경험, 자살 생각, 스트레스 인지 경험 수준이 더 높았다. 그러나 코로나 19 이전부터 정신건강이 더 취약했던 하위 그룹이 코로나19의 유행에 더 큰 영향을 받지는 않았다.

코로나 19 유행 확산을 막기 위해 학교가 폐쇄되고 학생들의 활동이 제한되면서 청소년들은 사회적으로 고립되었는데, 몇몇 선행연구에서는 이러한 일상생활 변화로 인해 유행 기간 중 우울, 불안, 자살 경향성 증가 등 정신건강이 악화되었다고 보고하였다[16,18,19]. 반면 미국 청소년을 대상으로 수행한 연구에서는 코로나19로 인해 학교에 가지 않거나 방과후 활동 및 운동을 하지 않게 되는 등의 일상생활 변화가 우울 증상이나 불안에 영향을 미치지 않은 것으로 보고하였다[14]. 본 연구에서는 코로나19 첫 번째 해에 한국 청소년이 경험한 우울감 경험, 자살 생각, 스트레스 인지율이 코로나19 이전에 비해 더 낮은 수준인 것으로 나타났다. 연구 결과 간에 상이한 결과가 나오는 것은 각 나라마다 거리두기 정책 및 청소년 생활 패턴과 문화가 다르기 때문일 가능성이 있다. 우리나라의 경우 2020년 1월 국내에 코로나19 첫번째 확진 환자가 발생하면서 코로나19 확산 방지를 위한 정책에 의해 2020년 1학기 개학이 연기되었고 4월부터 학년에 따라 순차적으로 온라인 수업과 등교가 병행되었다. 2020년 청소년건강행태조사는 8~11월에 시행되었으므로 2020년 조사 결과에는 개학이 연기되다가 온라인 수업이 병행되는 시점의 청소년 정신건강 상태가 반영되었을 것이다. 즉, 본 연구 결과는 코로나19 유행 첫번째 해에는 학교 폐쇄 및 온라인 수업으로 인해 친구들과의 관계 및 학업에서 오는 스트레스가 일시적으로 줄어들고, 수면충족률이 증가하고 가족들과 보내는 시간이 증가하여 우울감, 자살생각, 스트레스 인지 정도가 감소했을 가능성을 시사한다[3].

반면 본 연구 결과에서 2021년의 우울감 경험, 자살 생각, 스트레스 인지율은 2020년에 비해 더 높았다. 9~24세 아동청소년 대상의 온라인 조사 결과에서도 우리 연구 결과와 유사하게 2020년에 비해 2021년에 코로나로 인한 불안, 분노, 두려움, 스트레스를 더 많이 경험하였고 관심, 감사와 같은 긍정적인 정서 경험은 감소한 것으로 나타났다. 또한 고위험 청소년의 경우 이러한 심리적 어려움이 더 심해진 것으로 조사되었다[20]. 2021년 청소년건강행태조사는 8~11월에 조사가 시행되었으므로 2021년 조사 결과에는 코로나19가 장기화되는 상황에서의 청소년의 정신건강 상태가 반영되었을 가능성이 있다. 우리나라의 경우 2020년 2학기부터는 코로나19 확진 현황에 따라 사회적 거리두기 단계가 조정되었고 그에 따라 학생들의 등교 원칙에 계속적인 변화가 있었다. 지역과 학교 여건에 따라 차이가 있었으나 대체로 2021년 1학기에 고등학교 3학년을 제외한 나머지 학생들은 격주 혹은 격일로 등교하여, 사회적 격리와 이로 인한 외로움, 불규칙한 생활 패턴이 지속되었다. 본 연구결과는 코로나19의 장기화와 그로인한 불확실한 상황 및 사회적 고립의 지속이 감염에 대한 두려움, 스트레스, 학업 및 진로에 대한 걱정 등 부정적인 정서를 심화시켰을 가능성을 시사한다. 코로나19 유행이 앞으로 더 지속되면 청소년의 정신건강에 더 큰 부정적 영향을 미칠 가능성이 있으며 따라서 유행이 장기화됨에 따라 청소년의 정신건강에 미치는 영향 및 그 관련요인에 관한 후속 연구가 필요하겠다.

코로나19 유행이 모든 인구집단에서 동일한 크기의 영향을 미치는 것이 아니며 더 큰 영향을 받는 취약계층이 있다는 것이 여러 연구에서 발표되었으며[4], 여학생, 낮은 교육 수준, 낮은 가정 경제 수준, 좁은 생활 공간, 기저 정신질환[5,8,21] 등의 특징을 갖는 인구집단에서 코로나19에 의한 영향을 더 크게 받는 것으로 보고되었다. 본 연구에서도 가족과 함께 살지 않는 경우, 가정 경제형편이 좋지 않은 경우, 코로나 19 이후 가정 경제 상태가 악화된 경우, 자신이 건강하지 않다고 인지하는 경우에 코로나19의 영향에 더 취약한 것으로 나타났다. 이러한 연구 결과는 코로나로 인한 정신건강의 질병부담을 줄이기 위해서는 취약집단을 대상으로 집중적인 중재가 이루어져야 함을 시사한다.

본 연구는 코로나19 이후 2년에 걸친 자료를 포함하여 분석함으로써 코로나19 유행의 지속이 청소년 정신건강에 미치는 영향을 살펴보았다는 점에서 이전에 수행된 대부분의 연구와 차이점이 있다. 또한 청소년건강행태조사는 대한민국 청소년을 대표할 수 있는 추정치를 산출할 수 있도록 추출된 표본을 대상으로 시행되었으며, 매년 같은 방법을 사용하여 조사하였기 때문에 년도에 따른 유병률 추세 비교도 할 수 있었다. 그러나, 여러 해의 자료를 이용하여 년도에 따른 추세를 분석하기는 하였으나 단면조사 자료를 바탕으로 분석하였기 때문에 개인 수준에서 시간에 따른 정신건강의 변화를 분석하지 못한 제한점이 있다. 또한 기존 정신건강 상태가 좋지 않은 경우에 코로나19 유행으로 인한 영향을 더 크게 받는 것으로 알려져 있음에도 불구하고[16], 본 연구에서는 기존 정신건강 상태에 대한 정보가 없어 이를 분석 시 고려하지 못하였다.

**Conclusion**

코로나19 유행 초기에는 청소년의 정신건강이 개선되는 경향이었으나 유행 상황이 지속되면서 우울감, 자살 생각, 스트레스 인지 경험이 유의하게 증가하였다. 또한 가족과 함께 살지 않는 경우, 가정 경제 상태가 낮은 경우, 자신의 건강상태가 건강하지 않다고 인지하는 경우에 대체적으로 우울감, 자살 생각, 스트레스 인지 경험이 더 높았다. 코로나19가 청소년 정신건강에 미치는 장기적인 영향에 대한 이해는 코로나19 유행 이후 청소년 정신건강 증진을 위한 공중보건학적 전략수립에 중요하다.

**Conflict of interests**

The authors have no conflicts of interest to declare for this study.

**Funding**

None

**Acknowledgements**

None

**Author’s contribution**

Conceptualization: Park B, Oh K. Data curation: Yang J, Kim J. Formal analysis: Yang J, Choi S. Methodology: Park B, Yang J. Project administration: Choi S, Oh K. Visualization: Park B, Kim J, Yang J. Writing – original draft: Park B, Kim J. Writing – review and editing: Park B, Kim J, Choi S, Oh K.

**ORCID**

Bomi Park 0000-0001-5834-9975

Jihee Kim 0000-0002-0212-8177

Jieun Yang 0000-0002-6469-6057

Sunhye Choi 0000-0002-2942-0290

Kyungwon Oh 0000-0001-8097-6078

**Key summary**

코로나19가 청소년 정신건강에 영향을 미친다는 선행 연구결과가 있었으나 이전 연구들은 자료의 크기가 작거나 코로나19의 초기 영향만을 살펴보았다. 본 연구에서는 대규모 건강조사를 이용하여 코로나19가 장기화됨에 따른 영향을 평가하고자 하였다. 2018년-2021년 청소년건강행태조사에 참여한 만 12-18세 청소년 227,139 명을 대상으로 분석한 결과, 코로나19 유행 첫번째 해(2020년)에는 코로나19 유행 이전에 비해 정신건강이 개선되었으나, 코로나19 유행 두 번째 해(2021년)에는 코로나19 유행 첫 번째 해에 비해 청소년 정신건강이 나빠졌다. 또한 가족과 함께 거주하지 않는 경우, 가정 경제 수준이 낮은 경우, 본인이 건강하지 않다고 인지하는 경우에 더 취약하였다. 이번 연구는 코로나19 장기화가 청소년의 정신건강에 부정적 영향을 미칠 가능성이 있다는 것과 코로나19 유행에 더욱 취약한 하위집단이 있음을 보여주었다.
