# Supplementary material for: Changes in mental health of Korean adolescents before and during the COVID-19 pandemic: a special report using the Korea Youth Risk Behavior Survey
Source: Epidemiol Health. 2023 Feb 14;45:e2023019. doi: 10.4178/epih.e2023019 (PMC10581890; doi:10.4178/epih.e2023019)
Supplement: Supplementary Material 1. — The prevalence of depressive symptoms, suicidal ideation, and stress perception from 2012 to 2021 in the Korea Youth Risk Behavior Survey [file epih-45-e2023019-Supplementary-1.docx]

Supplementary Material 1. The prevalence of depressive symptoms, suicidal ideation, and stress perception from 2012 to 2021 in the Korea Youth Risk Behavior Survey

|  | Before COVID-19 | | | | | | | |  | During COVID-19 | |
| --- | --- | --- | --- | --- | --- | --- | --- | --- | --- | --- | --- |
|  | 2012 | 2013 | 2014 | 2015 | 2016 | 2017 | 2018 | 2019 |  | 2020 | 2021 |
| Depressive symptoms |  |  |  |  |  |  |  |  |  |  |  |
| Total | 30.5 (0.2) | 30.9 (0.3) | 26.7 (0.2) | 23.6 (0.2) | 25.5 (0.2) | 25.1 (0.2) | 27.1 (0.3) | 28,2 (0.3) |  | 25.2 (0.3) | 26.8 (0.2) |
| Sex |  |  |  |  |  |  |  |  |  |  |  |
| Male | 25.5 (0.3) | 25.2 (0.3) | 22.2 (0.2) | 19.7 (0.2) | 20.9 (0.2) | 20.3 (0.3) | 21.1 (0.3) | 22.2 (0.3) |  | 20.1 (0.3) | 22.4 (0.3) |
| Female | 36.0 (0.3) | 37.1 (0.3) | 31.6 (0.3) | 27.8 (0.3) | 30.5 (0.3) | 30.3 (0.3) | 33.6 (0.3) | 34.6 (0.4) |  | 30.7 (0.3) | 31.4 (0.3) |
| Grade |  |  |  |  |  |  |  |  |  |  |  |
| Middle school | 28.2 (0.3) | 29.0 (0.3) | 24.4 (0.3) | 21.2 (0.3) | 22.7 (0.3) | 23.5 (0.3) | 25.2 (0.4) | 26.9 (0.4) |  | 22.9 (0.3) | 25.9 (0.3) |
| High school | 32.6 (0.3) | 32.6 (0.4) | 28.8 (0.3) | 25.6 (0.3) | 27.7 (0.4) | 26.4 (0.4) | 28.7 (0.4) | 29.4 (0.4) |  | 27.4 (0.4) | 27.7 (0.4) |
| Residential areas |  |  |  |  |  |  |  |  |  |  |  |
| Urban | 30.5 (0.3) | 31.3 (0.4) | 26.9 (0.3) | 23.3 (0.3) | 25.1 (0.4) | 24.8 (0.3) | 27.0 (0.4) | 27.6 (0.4) |  | 24.4 (0.4) | 25.7 (0.4) |
| Rural | 30.5 (0.3) | 30.5 (0.4) | 26.6 (0.3) | 23.8 (0.3) | 25.7 (0.3) | 25.3 (0.3) | 27.1 (0.4) | 28.6 (0.4) |  | 25.8 (0.3) | 27.5 (0.3) |
| Suicidal ideation |  |  |  |  |  |  |  |  |  |  |  |
| Total | 18.3 (0.2) | 16.6 (0.2) | 13.1 (0.2) | 11.7 (0.1) | 12.1 (0.2) | 12.1 (0.2) | 13.3 (0.2) | 13.1 (0.2) |  | 10.9 (0.2) | 12.7 (0.2) |
| Sex |  |  |  |  |  |  |  |  |  |  |  |
| Male | 14.1 (0.2) | 13.1 (0.2) | 11.0 (0.2) | 9.6 (0.2) | 9.5 (0.2) | 9.4 (0.2) | 9.6 (0.2) | 9.4 (0.2) |  | 8.1 (0.2) | 9.5 (0.2) |
| Female | 22.9 (0.3) | 20.4 (0.3) | 15.4 (0.2) | 13.9 (0.2) | 14.9 (0.2) | 15.0 (0.2) | 17.4 (0.3) | 17.1 (0.3) |  | 13.9 (0.3) | 16.1 (0.3) |
| Grade |  |  |  |  |  |  |  |  |  |  |  |
| Middle school | 18.5 (0.3) | 17.6 (0.3) | 13.3 (0.2) | 11.7 (0.2) | 12.0 (0.3) | 12.7 (0.2) | 13.8 (0.3) | 14.1 (0.3) |  | 10.2 (0.2) | 13.4 (0.3) |
| High school | 18.0 (0.3) | 15.6 (0.2) | 12.8 (0.2) | 11.6 (0.2) | 12.2 (0.2) | 11.6 (0.2) | 12.9 (0.3) | 12.2 (0.2) |  | 11.5 (0.3) | 12.0 (0.3) |
| Residential areas |  |  |  |  |  |  |  |  |  |  |  |
| Urban | 18.5 (0.3) | 16.9 (0.3) | 13.4 (0.2) | 11.7 (0.2) | 12.0 (0.3) | 12.3 (0.2) | 13.3 (0.3) | 12.9 (0.3) |  | 10.5 (0.2) | 12.1 (0.3) |
| Rural | 18.1 (0.3) | 16.3 (0.3) | 12.8 (0.2) | 11.7 (0.2) | 12.2 (0.2) | 11.9 (0.2) | 13.3 (0.2) | 13.3 (0.2) |  | 11.2 (0.2) | 13.2 (0.2) |
| Stress perception |  |  |  |  |  |  |  |  |  |  |  |
| Total | 41.9 (0.3) | 41.4 (0.3) | 37.0 (0.3) | 35.4 (0.3) | 37.4 (0.3) | 37.2 (0.3) | 40.4 (0.3) | 39.9 (0.3) |  | 34.2 (0.3) | 38.8 (0.3) |
| Sex |  |  |  |  |  |  |  |  |  |  |  |
| Male | 34.8 (0.3) | 34.3 (0.3) | 30.8 (0.3) | 29.6 (0.3) | 30.5 (0.3) | 30.4 (0.3) | 32.0 (0.3) | 31.7 (0.3) |  | 28.1 (0.3) | 32.3 (0.3) |
| Female | 49.6 (0.4) | 49.3 (0.3) | 43.7 (0.3) | 41.7 (0.4) | 44.9 (0.4) | 44.6 (0.4) | 49.5 (0.4) | 48.8 (0.4) |  | 40.7 (0.4) | 45.6 (0.4) |
| Grade |  |  |  |  |  |  |  |  |  |  |  |
| Middle school | 38.9 (0.4) | 39.1 (0.4) | 33.4 (0.4) | 31.6 (0.3) | 33.6 (0.3) | 34.1 (0.3) | 37.0 (0.4) | 37.2 (0.4) |  | 30.4 (0.4) | 36.4 (0.4) |
| High school | 44.7 (0.5) | 43.7 (0.5) | 40.3 (0.4) | 38.7 (0.4) | 40.5 (0.4) | 39.8 (0.5) | 43.4 (0.5) | 42.4 (0.5) |  | 37.9 (0.4) | 41.2 (0.5) |
| Residential areas |  |  |  |  |  |  |  |  |  |  |  |
| Urban | 41.7 (0.4) | 41.0 (0.4) | 36.9 (0.4) | 35.1 (0.4) | 36.7 (0.5) | 37.2 (0.5) | 40.2 (0.5) | 39.4 (0.5) |  | 33.5 (0.5) | 38.4 (0.4) |
| Rural | 42.0 (0.4) | 41.8 (0.4) | 37.0 (0.3) | 35.6 (0.4) | 37.8 (0.4) | 37.1 (0.4) | 40.6 (0.5) | 40.3 (0.4) |  | 34.6 (0.4) | 39.0 (0.4) |

Numbers are presented as weighted % (SE).
